# A Rapid and Precise Method for Nitrate Determination using a Step-Flow Autoanalyzer with Online Stopover Vanadium Reduction

**DOI:** 10.1021/acsomega.5c07258

**Published:** 2025-12-05

**Authors:** Su-Cheng Pai, Shun-Kai Chang, Chia-Te Chien, Tung-Yuan Ho

**Affiliations:** † Institute of Oceanography, 33561 National Taiwan University, Taipei 106319, Taiwan; ‡ Research Center for Environmental Changes, Academia Sinica, Taipei 115024, Taiwan

## Abstract

This study presents the development and performance evaluation of a Step-Flow Autoanalyzer that integrates online vanadium­(III) reduction with the Griess assay for the high-precision and efficient determination of dissolved nitrate in aqueous samples. The system manifold comprises seven microperistaltic pumps controlled by six electric relay modules. The nitrate sample is loaded, mixed with reagents, and held in a heating coil at 90 °C for 70 s to facilitate the reduction of nitrate to nitrite. It then passes through a cooling bath, lowering the temperature to below 60 °C to stabilize the pink azo dye color before being delivered to a 1 cm dome-type flow cuvette installed in a spectrophotometer. Absorbance at 543 nm is measured under static conditions. High precision is achieved (<0.5% RSD at the 10 μM level), with a detection limit of 0.1 μM, equivalent to 0.003 absorbance units (AU). The calibration curve remains nearly linear up to 50 μM, covering the concentration range of most environmental samples. The apparent reduction efficiency (E%), estimated by comparing responses from equivalent concentrations of nitrite and nitrate, is approximately 99% in freshwater and 92% in seawater. Each measurement cycle takes 120 s, allowing for a maximum throughput of 25–30 samples per hour. The system’s high precision and reliability during extended operation make it well-suited for routine applications involving large numbers of samples.

## Graphical abstract

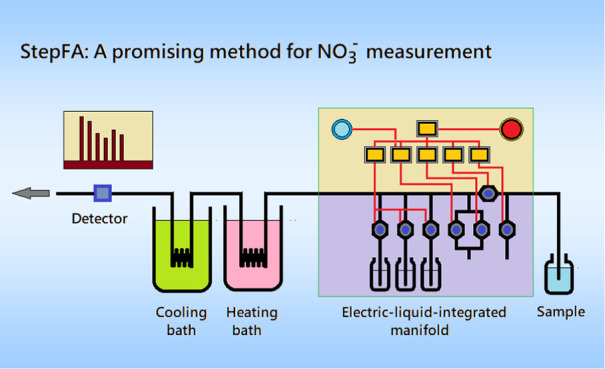

## Introduction

Nitrate is a key parameter in nearly all environmental studies, yet achieving the necessary precision and accuracy in its measurement remains challenging. For decades, nitrate has been measured using the cadmium reduction method, in which it is first reduced to nitrite and then reacts with Griess reagents to form a pink azo dye for colorimetric detection.^1,2^ This procedure is typically carried out using automated instruments such as flow injection analyzers (FIA)^3–7^ or programmable flow injection (pFI),^8^ which employ an online copper-coated cadmium column. Despite various improvements and modifications, significant uncertainties remain—primarily due to the difficulty of preparing and maintaining a cadmium column with consistent reduction efficiency.^9,10^ Moreover, the environmental toxicity of cadmium makes its continued use increasingly undesirable.

To address these issues, vanadium­(III) reduction has emerged as a safer alternative.^11,12^ Vanadium­(III) can be prepared in liquid form and is significantly less toxic than cadmium. However, its reduction reaction proceeds relatively slowly and requires elevated temperatures to achieve acceptable efficiency. Unfortunately, heating also accelerates the fading of the pink azo dye, creating a trade-off between reaction efficiency and color stability. Although manual procedures using vanadium­(III) have been described, they are often tedious and time-consuming.^13–17^ Several studies have attempted to implement online vanadium reduction using conventional flow injection analysis (FIA) systems^18,19^ and sequential injection analyzer (SIA).^20^ However, the reduction achieved with these techniques was likely incomplete. For instance, in the study employing SIA,^20^ a syringe pump transferred the sample–reagent mixture into a heating coil at 70 °C for 90 s, after which it was delivered into a flow cuvette for stop-flow detection. All peaks showed a rising trend at the apex, and the apparent reduction efficiency was only 65%, as indicated by the calibration slopes of 0.0342 μM^–1^ for nitrite and 0.0221 μM^–1^ for nitrate. This highlights the need for further optimization, particularly of the heating process.

A recently developed automated system, the Step-Flow Autoanalyzer (StepFA),^21^ was designed for the colorimetric analysis of nitrite, phosphate, and silicate, and is well-suited for adaptation to nitrate measurements. The StepFA employs micro peristaltic pumps, with flow paths and timing precisely controlled by electric relay modules. In this study, we present a newly designed StepFA manifold specifically optimized for nitrate determination via vanadium­(III) reduction. The system retains the sample segment in an online heating coil for a defined duration, allowing sufficient time for the reduction reaction to proceed. This is followed by an online cooling step to stabilize the pink azo dye. As a result, high nitrate reduction efficiency and precise, accurate quantification are achieved.

## Experimental Section

### Instrument Layout

The proposed manifold for nitrate is based on a previously described StepFA design,^21^ with several modifications to accommodate the significantly increased in-tube pressure resulting from the addition of incubation and cooling coils. The manifold is illustrated in Figure 1. It consists of seven 24 V DC micro peristaltic pumps (P1–P7, Kamoer NKP-SA06), six integrated electric relays (T, L, R, A, D, and F), an online coil immersed in a 1 L heating bath, another online coil placed in a 1 L cooling bath, and a spectrophotometer (Metertech, Taiwan) equipped with a 1 cm dome-type flow cuvette (Hellma, Germany) featuring a 0.45 × 1 cm window and a capacity of 450 μL. The micro pumps operate in instant, delayed, or loop mode, controlled by preset relay programs to drive liquid flow in a stepwise manner.

**Figure 1:**
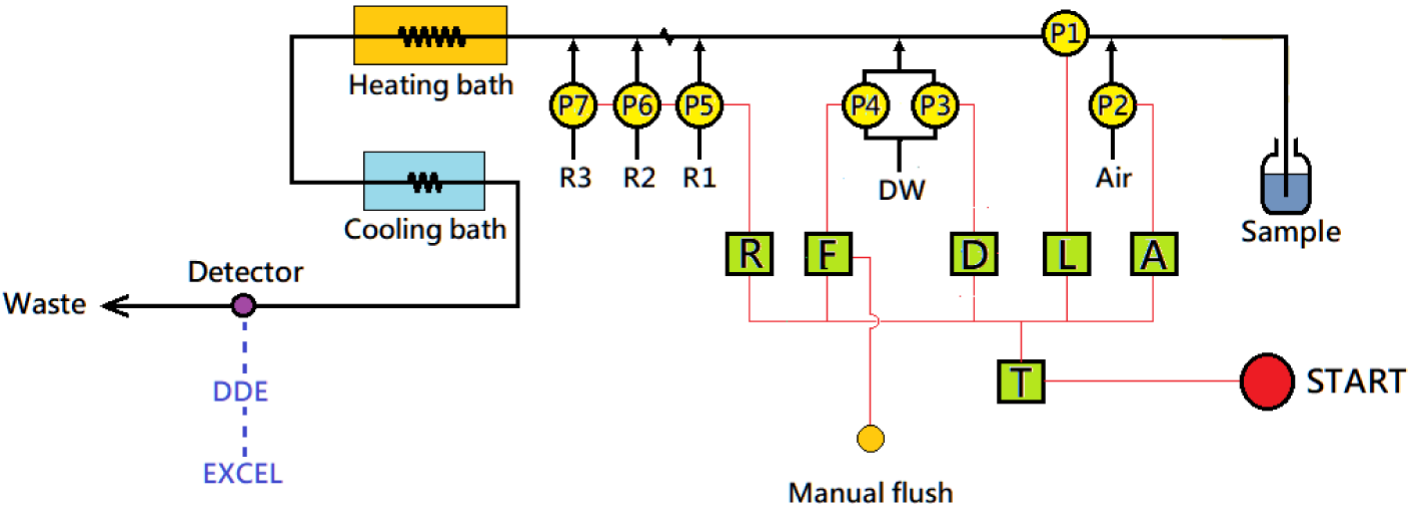
Layout of the step-flow manifold for nitrate determination. The system consists of six electric relay modules (labeled T, L, A, D, F, and R), seven micro peristaltic pumps (labeled P1–P7), a 1-L heating bath, a 1-L water-filled cooling bath, and a detector unit equipped with a wide-bore dome-top flow cuvette. Signals are transmitted via a DDE device and processed by an Excel worksheet for real-time display. (Black lines) Liquid tubing; (Red lines) Electric control wires; (Blue dotted lines) Signal transmission.

The flow rate of each micro peristaltic pump is determined by the size of the pumping tube and the applied DC voltage. Pumps P1, P2, P3, and P4 are fitted with 2 mm ID (internal diameter) silicone rubber tubes and powered by 24 V DC. Reagents are delivered by pumps P5, P6, and P7 using 0.8 mm ID BPT tubing (biopharmaceutical-grade, Saint-Gobain, France) and powered by 7.5 V DC. The complete configuration and relay timing settings are detailed in Table 1.

**Table 1:** Settings of Relays and Pumps of a 120 s Measuring Cycle for NO_3_^–^ Measurement

**Function**	**Relay**	**Voltage (DCV)**	**Pump**	**Tuble ID (mm)**	**Operation mode**	**Action period**
Trigger	T	24			Loop^a^	1s
Load sample	L	24	P1	2	Instant	0–10s
Add reagent R1	R	7.5	P5	0.8	Delay	2–10s
Add reagent R2	R	7.5	P6	0.8	Delay	2–10s
Add reagent R3	R	7.5	P7	0.8	Delay	2–10s
Air injection/reflux	A	24	P2	2	Delay	9–11s
Deliver to detector	D	24	P3	2	Delay	80–90s
Flush with DW	F	24	P4	2	Delay	105–120s

a The loop mode allows single or repeating measurements.

To initiate the measurement, the “start” button was pressed to trigger Relay T, which simultaneously activated the other relays (L, R, A, D, and F). Each relay controlled its respective pump(s) according to the programmed sequence. In a typical 120-s cycle: Relay L operated pump P1 to load the sample from 0 to 10 s; Relay R activated pumps P5, P6, and P7 to introduce reagents into the main flow stream from 2 to 10 s; Relay A controlled pump P2 to draw air from 9 to 11 s, creating air segmentation and inducing reflux to clear residual sample; Relay D operated pump P3 from 80 to 90 s to deliver the reacted sample through the cooling bath and into the detector. After the maximum absorbance signal was recorded, Relay F activated pump P4 to flush the main tubing from 105 to 120 s. The effective incubation time for the sample within the heating coil was 70 s.

### Reagents

Three reagents were prepared separately and added online at equal pumping rates:

R1 [HSUL]: dissolve 6 g of sulfanilamide (Thermo Scientific, mol. wt. = 172.2 g·mol^–1^) in 100 mL of 15% (v/v) HCl.

R2 [NED]: dissolve 0.15 g of N-1-naphthylethylenediamine dihydrochloride (Sigma-Aldrich, mol. wt. = 259.18 g·mol^–1^) in 100 mL of distilled water.

R3 [VCl3]: dissolve 3.2 g of vanadium­(III) chloride (Thermo Scientific, mol. wt. = 157.3 g·mol^–1^) in 100 mL of 15% (v/v) HCl. This reagent was prepared in a fume cupboard to avoid inhalation hazards. The freshly prepared VCl_3_ solution is dark blue, may appear slightly cloudy, and should be filtered before use.

### Data Acquisition and Processing

The signal from the spectrophotometer was acquired directly via Dynamic Data Exchange (DDE) through an RS-232 port to a computer at a rate of one data point per second. The signal was recorded in an Excel worksheet, enabling real-time visualization of absorbance traces (peaks) on the screen. After each measurement cycle, or every 50 s, the software checked the baseline and automatically verified and corrected it to compensate for any baseline drift. Large bubble spikes were filtered using a VBA script within the Excel worksheet. Further details of the data acquisition and processing procedures are consistent with those described in a previous study.^21^

### Reduction Efficiency

An apparent reduction efficiency E% for the vanadium­(III) process can be obtained by comparing measured absorbances for pure nitrite and nitrate standards at equal concentrations:

$$E\%(\mathrm{apparent})=[\mathrm{Abs}(N{O_{3}}^{‐})-\mathrm{RB}]/[\mathrm{Abs}(N{O_{2}}^{‐})-\mathrm{RB}]\times 100\%$$

Where RB is the reagent blank.

## Results and Discussion

### Strategy for Optimizing the Throughput

The capability of vanadium­(III) reduction has been discussed in many previous papers,^11–17^ but reported reduction efficiencies vary widely. This variability is mainly due to differences in the rates of color formation and fading of the pink azo dye, which originates from both initially present nitrite and nitrite produced during reduction—especially at higher temperatures.

In our previous manual method,^17^ samples were incubated at 50 °C for 25–30 min, then rapidly cooled in an ice bath to halt both the reduction reaction and color fading. Under these conditions, nitrite and nitrate exhibited an equal molar extinction coefficient of approximately 50,000 M^– 1^ cm^– 1^ in freshwater and 5-fold diluted seawater. However, the long incubation time is unsuitable for automated analysis, where faster throughput is required. To achieve this, a much higher reaction temperature is necessary.

In this study, we aimed to customize the StepFA design to reduce the total analysis time to 2 min while maximizing reduction efficiency and maintaining high precision and sensitivity. Four key factors were considered in the optimization process: incubation temperature, incubation time, vanadium concentration, and salinity. These variables may also interact with one another, adding complexity to the optimization. In addition, several secondary factors—such as reagent blank levels and cooling efficiency—should not be overlooked, as each can subtly affect the system’s performance and reliability. These factors are examined in the following sections.

### Tube Length and Timing

Precise timing of operations is critical for the effective implementation of the StepFA manifold. To verify sample movement along the tubing, a dense blue dye was used to visually track the positions of the sample, air, and flush water segments. The entire tubing was divided into 15 sections, spanning from the sample loading tip to the waste tank outlet (Figure 2). For example, the length from position 1 (loading tube tip) to position 2 (T-joint where air is injected) was 30 cm; from position 2 to the pumping tube was 3 cm; and the pumping tube of pump P1 between positions 3 and 4 measured 10 cm, and so forth. The total tubing length exceeded 7 m, with a tubular channel volume of approximately 13.5 mL, including the volumes of the pumping tubes, T-joints, and the flow cuvette.

**Figure 2:**
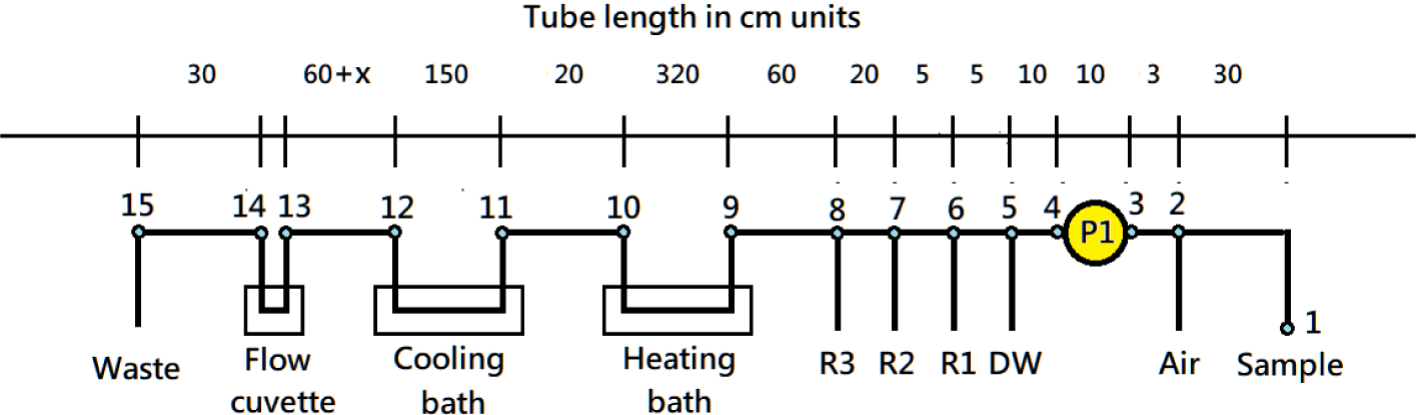
Schematic diagram illustrating the length of the main tubular channel from the loading tip to the waste outlet, divided into 14 sections by 15 numbered positions (1–15). A 1.5 mm ID Teflon tube was used for the main stream (except for the pumping tube at position P1). The length of each section is given in centimeters. The length between positions 12 and 13 was 60 + x cm, where “x” is an adjustable length to account for volume changes of air and liquid inside the tubing at different temperatures.

To aid reader understanding, the 120-s operation cycle is described into 10 consecutive stages (Figure 3):

**Figure 3:**
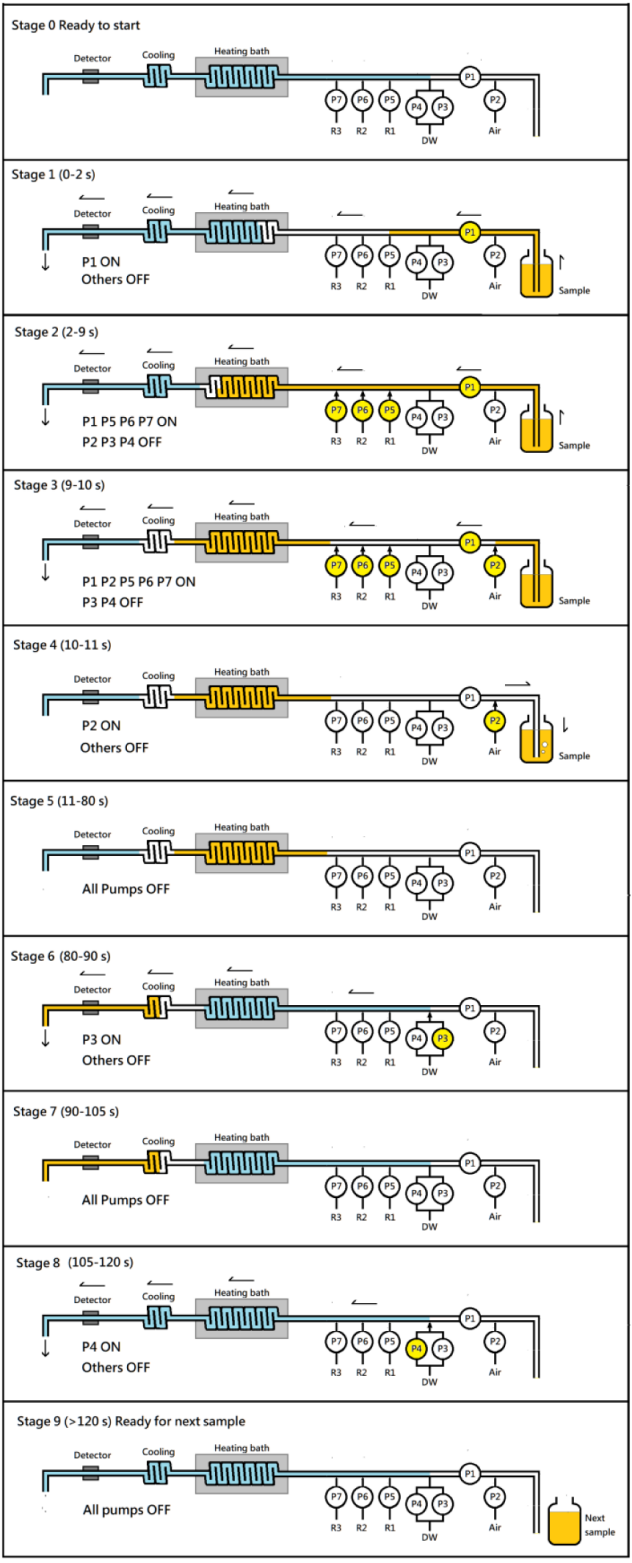
Schematic diagram illustrating fluid flow within the main tubing over 10 consecutive stages during a 120-s measurement cycle. Color codes indicate fluid segments: orange for the sample, blue for distilled water, and blank for air. Detailed descriptions of each stage are provided in the main text.

#### Stage 0: Before Starting

The system was run several times with distilled water to ensure that all reagent liquid fronts had reached the T-joints at the main flow (positions 6–8), and that distilled water filled the tubing from positions 5–15, while air occupied positions 1–5. The spectrophotometer absorbance was then set to zero.

#### Stage 1 (0–2 s)

After pressing the start button, pump P1 rapidly loaded the sample from the tip of the loading tube, advancing the sample front to position 6.

#### Stage 2 (2–9 s)

Pump P1 continued running, while reagent pumps P5, P6, and P7 were activated to merge reagents into the main stream. The sample front advanced to position 10.

#### Stage 3 (9–10 s)

Pumps P1, P5, P6, and P7 remained active, while pump P2 was turned on to inject a small air section passing through P1, reaching position 8. Simultaneously, the sample front moved to position 11.

#### Stage 4 (10–11 s)

All pumps stopped except pump P2, which continued for one more second to push residual sample remaining in the loading tube (positions 1–2) back to the original sample bottle. After this reflux process, air occupied space from position 1–8.

#### Stage 5 (11–80 s)

All pumps were stopped. The sample section occupied tubing from position 8–10 and remained there for incubation. The effective incubation time was 70 s.

#### Stage 6 (80–90 s)

Pump P3 was activated for 10 s, delivering the sample through the cooling bath and the detector. When the air section passed the detector, it caused a first bubble spike at ca. 83 s. The sample section occupied position from 11 to 15.

#### Stage 7 (90–105 s)

All pumps stopped. The sample remained statically trapped in the flow cuvette for 15 s while the detector recorded a steady absorbance signal, and a peak value was identified at ∼100 s.

#### Stage 8 (105–120 s)

Pump P4 was automatically activated for flushing, pushing distilled water from position 5-15 for 15 s. A second air section passed through the detector at ∼108 s, causing another bubble spike. When flushing stopped, the absorbance returned to zero.

#### Stage 9 (≥120 s)

The manifold returned to its initial state, with air occupying positions 1–5 and distilled water from position 5–15, ready for the next sample.

### Temperature Control

The 1 L heating bath was set to 90 °C, although the actual temperature inside the coil likely did not reach this set point. After stop-over incubation, the heated sample was delivered to the detector through a 1 L water-filled cooling bath. While the water temperature in the cooling bath gradually increased during operation, but not exceeding 40 °C. The temperature at the outlet of the flow cuvette (position 15 in Figure 2) was measured to fluctuate between 37–55 °C during consecutive sample loading, delivery, and flushing stages. Based on these observations, the sample temperature inside the cuvette during detection was estimated to be around 55 °C. The pink azo dye color was quite stable within 1 min at this temperature.

### Pumping Rates

The actual pumping rate of each individual pump was estimated by measuring the weight loss at the sample loading tip and the weight gain at the outflow end, with the air pump turned off. In a typical 10-s loading process, the sample lost 7.55 g in weight, corresponding to a pumping rate of 45.3 mL min^–1^ for pump P1. Simultaneously, the liquid collected at the end of the manifold weighed 8.91 g, reflecting both the 10-s sample loading and the 8-s reagent addition. Based on this, the pumping rate for each reagent pump was estimated to be 3.4 mL min^–1^. During the 10-s delivery period, the outflow weighed 6.98 g, giving pump P3 a flow rate of 41.9 mL min^–1^. Similarly, during the 15-s flushing period, the outflow weighed 11.27 g, corresponding to an estimated flow rate of 45.1 mL min^–1^ for pump P4.

When the air pump resumed operation, the actual sample uptake was 6.16 mL. The volume of the first air segment was estimated at 1.4 mL, and the second air segment was approximately 0.5 mL. During a 120-s measurement cycle, a total of 25.4 mL of waste liquid was generated, consisting of 6.16 mL of sample, 1.36 mL of reagents, 17.9 mL of flush water, and approximately 2 mL of air segments.

### Signal- and Spike-Filtering

Typical nitrate signals obtained using the StepFA method are shown in Figure 4. Each raw signal peak exhibits a flat top, indicating that the colorimetric reaction has either completed or ceased. Three main types of spikes are generally observed:

(a) Small spikes caused by the passage of tiny bubbles generated in the heating coil. These usually last only 1–2 s, with random signal heights that rarely exceed 0.030 AU.
(b) Large spikes caused by the passage of a large air segment (∼1.4 mL) ahead of the sample section. These often include Schlieren effects resulting from temperature or matrix gradients between the sample and the flushing liquid. They typically last 2–3 s, with signal amplitudes well exceeding 0.200 AU.
(c) End-of-sample spikes caused by the passage of another air segment (∼0.5 mL) at the end of the sample section, again accompanied by temperature and matrix gradient effects. These spikes might be partially masked by the sample signal if the sample absorbance is higher than the spike value.

**Figure 4:**
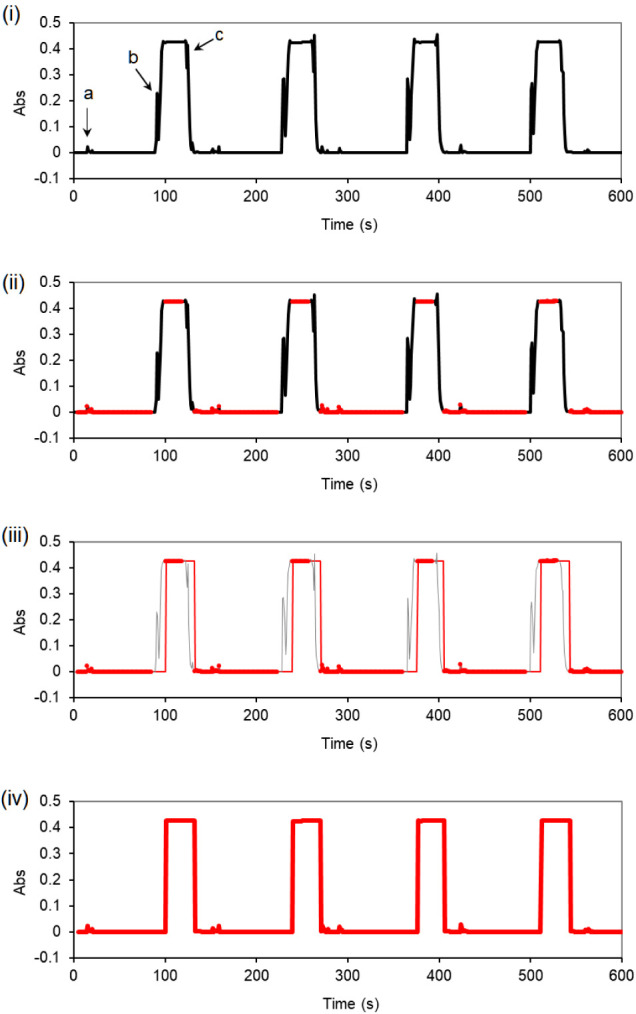
Typical peak shapes for nitrate measurement using the StepFA system. (i) The raw signal typically exhibits three types of spikes, labeled a, b, and c. Small spikes (type-a) were left untreated, while large spikes (types-b and c) need to be removed. (ii) A gradient-based software filter is applied using the criterion “ABS­(d*A*/d*t*) > 0.04” to detect sudden absorbance changes exceeding 0.040 AU per second. Data points within ± 5 s of each detected change are considered invalid. Valid data are highlighted in red, while invalid data points are marked in black. (iii) For excluded (invalid) data points, the software automatically fills values using the last valid measurement, i.e., Abs­(t) = Abs­(t–1). (iv) The filtered peaks appear as blocky histograms on the display screen.

All spikes occur only during liquid flow—no spike signals appear when the flow is stopped. Small spikes (type-a) may be left untreated, but large bubble spikes (type-b and type-c), which may confuse analysts, must be filtered. In the Excel worksheet, once the real-time signal is acquired, a Visual Basic for Applications (VBA) script is applied using the formula “=IF­(ABS­(d*A*/d*t*)>0.04,1,0)” to detect sudden absorbance changes greater than 0.040 AU. Data points within ± 5 s of these spikes are flagged as invalid. In other words, any spike exceeding 0.040 AU, along with its neighboring data points, is automatically erased and replaced with the last valid data. As a result, the processed signal appears as a blocky histogram, representing the steady-state value of each measurement.

### Incubation Temperature

An ordinary 1-L thermostatic teapot was used as the heating bath for sample incubation, the actual bath temperature was monitored by a thermometer, showing a variation of ±2 °C during a measuring cycle. For this experiment, reagent blanks, as well as 10 μM nitrite and nitrate standard solutions, were prepared in both distilled water and seawater media. The seawater sample, depleted of nitrite and nitrate, was collected from the surface during a cruise to the South China Sea. The bath temperature was varied from room temperature to 95 °C, and the results are summarized in Figure 5. All measurements shown in the figure were performed in at least triplicate, yielding an overall precision better than 0.5% for nitrite measurements at all temperatures, and for nitrate at temperatures above 60 °C.

**Figure 5:**
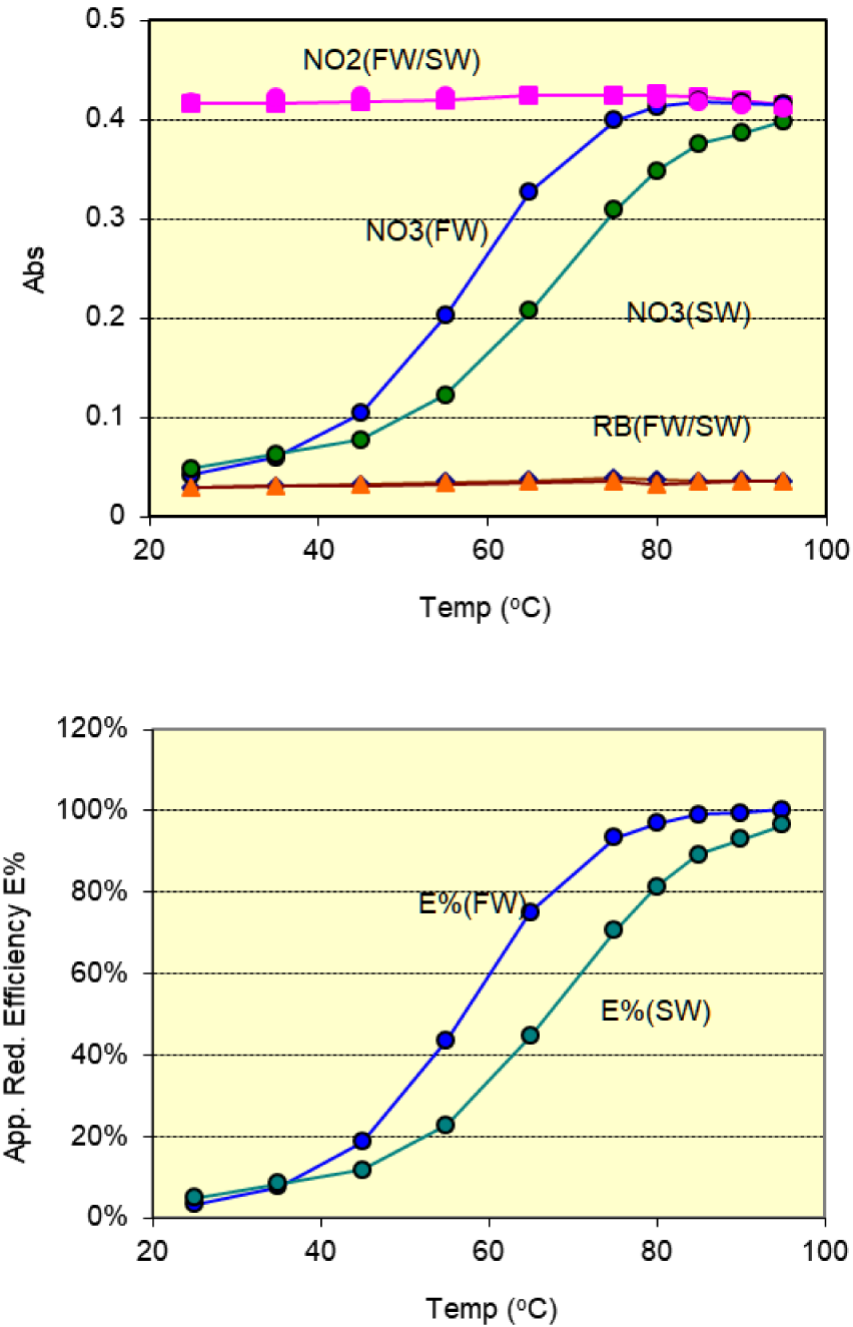
(Up) effect of incubation temperature on the absorbance of reagent blank, nitrite, and nitrate standard solutions, and (down) the apparent reduction efficiency (*E*%). The incubation duration was fixed at 70 s. Colors represent: pink – 10 μM nitrite in freshwater and seawater; blue – 10 μM nitrate in freshwater; green – 10 μM nitrate in seawater; orange – reagent blank.

The reagent blank showed a slight increasing trend with temperature, to be 0.035 AU at room temperature and 0.040 AU at 95 °C. For nitrite solutions, the formation of pink azo dye was obviously complete at incubation temperatures higher than 50 °C after a 70-s incubation period, with no noticeable difference between freshwater and seawater media. However, a slight decrease in absorbance was observed above 80 °C, indicating the onset of fading of pink azo dye.

The results for nitrate exhibited marked differences between the two media. In freshwater, the apparent reduction efficiency (E%) was 97% at 80 °C, increased to 98% at 85 °C, and reached >99% at 90 and 95 °C. In seawater, however, the efficiency was significantly lower: 81% at 80 °C, increasing to 88% at 85 °C, 92% at 90 °C, and reaching 95% at 95 °C. The exact reason for the matrix effect has not been identified, it may be related to the high chloride content in seawater and the presence of other competing ions.

Although the reduction efficiency at 95 °C was the highest, other factors needed to be considered: the fading of the pink azo dye was more pronounced, cooling was less effective, and more tiny bubbles formed in the heating coil. As a result, 90 °C was chosen for the incubation.

### Vanadium Concentration

The concentration of vanadium is also a critical factor in the nitrate reduction process. In our previous manual method,^17^ a final VCl_3_ concentration of 10.18 mM was recommended; however, this concentration resulted in a relatively high reagent blank of approximately 0.050 AU. In the present study, we evaluated whether the vanadium concentration could be lowered without compromising reduction efficiency or overall analytical performance.

A series of VCl_3_ solutions were prepared by initially dissolving 6.4 g of vanadium (III) chloride in 100 mL of 15% (v/v) HCl, yielding a 6.4% (w/v) stock solution. After ultrasonic shaking and disc filtration, this stock was diluted with 15% (v/v) HCl to obtain working solutions at concentrations of 0.1%, 0.2%, 0.4%, 0.8%, 1.6%, 3.2%, and 4.8%(w/v). These reagents were tested with both 10 μM nitrite and nitrate standard solutions under controlled incubation conditions of 90 °C for 70 s. The resulting data are presented in Figure 6.

**Figure 6:**
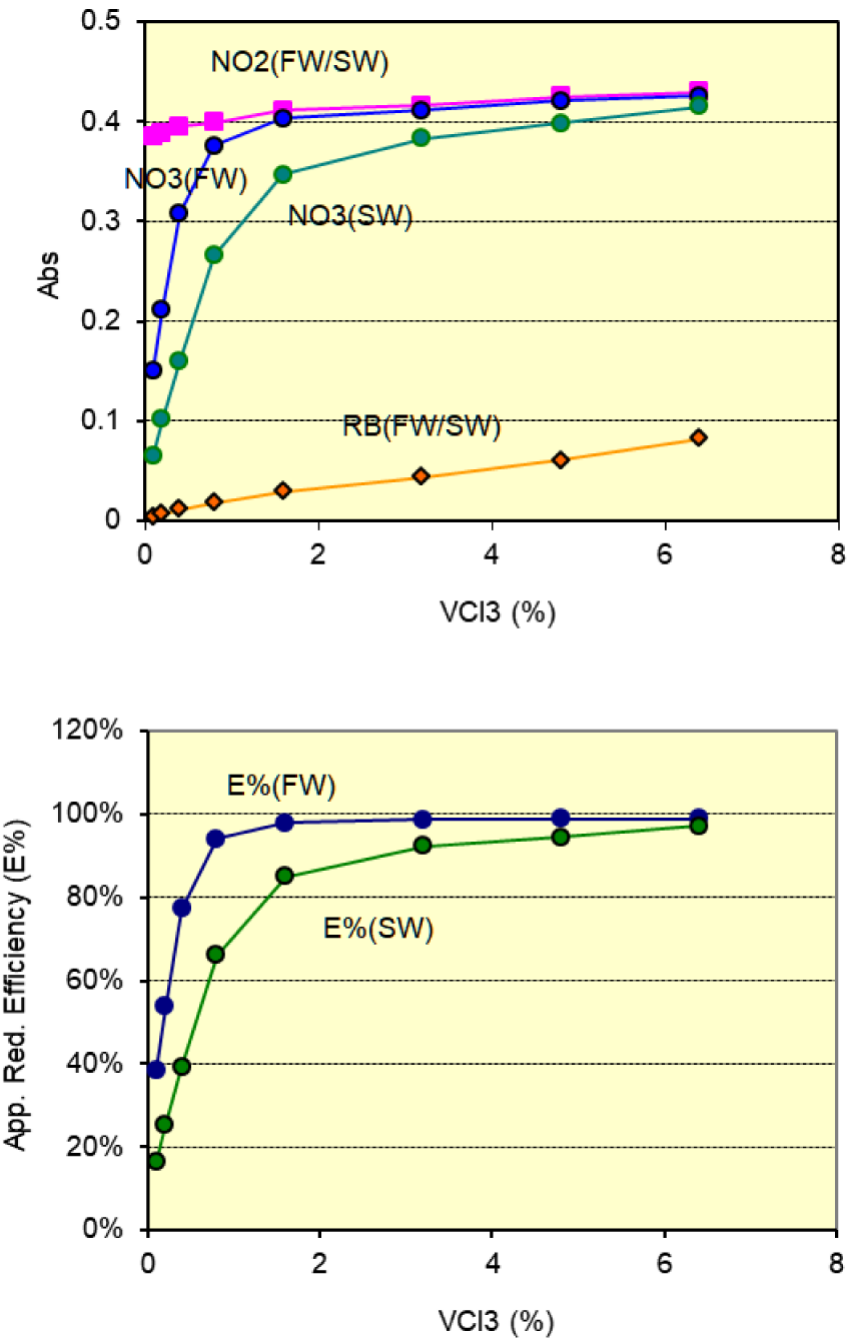
Effect of vanadium reagent concentration on (up) the absorbance of reagent blank, nitrite, and nitrate standard solutions, and (down) the apparent reduction efficiency (*E*%). Colors represent: pink – 10 μM nitrite in freshwater and seawater; blue – 10 μM nitrate in freshwater; green – 10 μM nitrate in seawater; orange – reagent blank.

It was observed that the reduction reaction was clearly incomplete when vanadium concentrations were below 1%. When concentrations exceeded 1.6%, the reduction efficiency increased and gradually approached an equilibrium. In freshwater medium, the 3.2% reagent achieved an apparent reduction efficiency approximately 99%, with a reagent blank of 0.040 AU. Further increasing the reagent strength to 6.4% resulted in almost the same efficiency, but the reagent blank was raised to 0.078 AU. In seawater, the reduction efficiency was 92% with a 3.2% reagent, 94% with a 4.8% reagent, and 98% with a 6.4% reagent. Considering the trade-off between reduction efficiency and reagent blank, the 3.2% vanadium solution was selected for all subsequent experiments.

### Incubation Time

The reagent blank, a 10 μM nitrite standard, and a 10 μM nitrate standard were incubated at 90 °C under varying incubation times. The net incubation time was defined as the interval between the stopping of pump P1 (end of sample loading) and the activation of pump P3 (start of sample delivery). The results are summarized in Figure 7.

**Figure 7:**
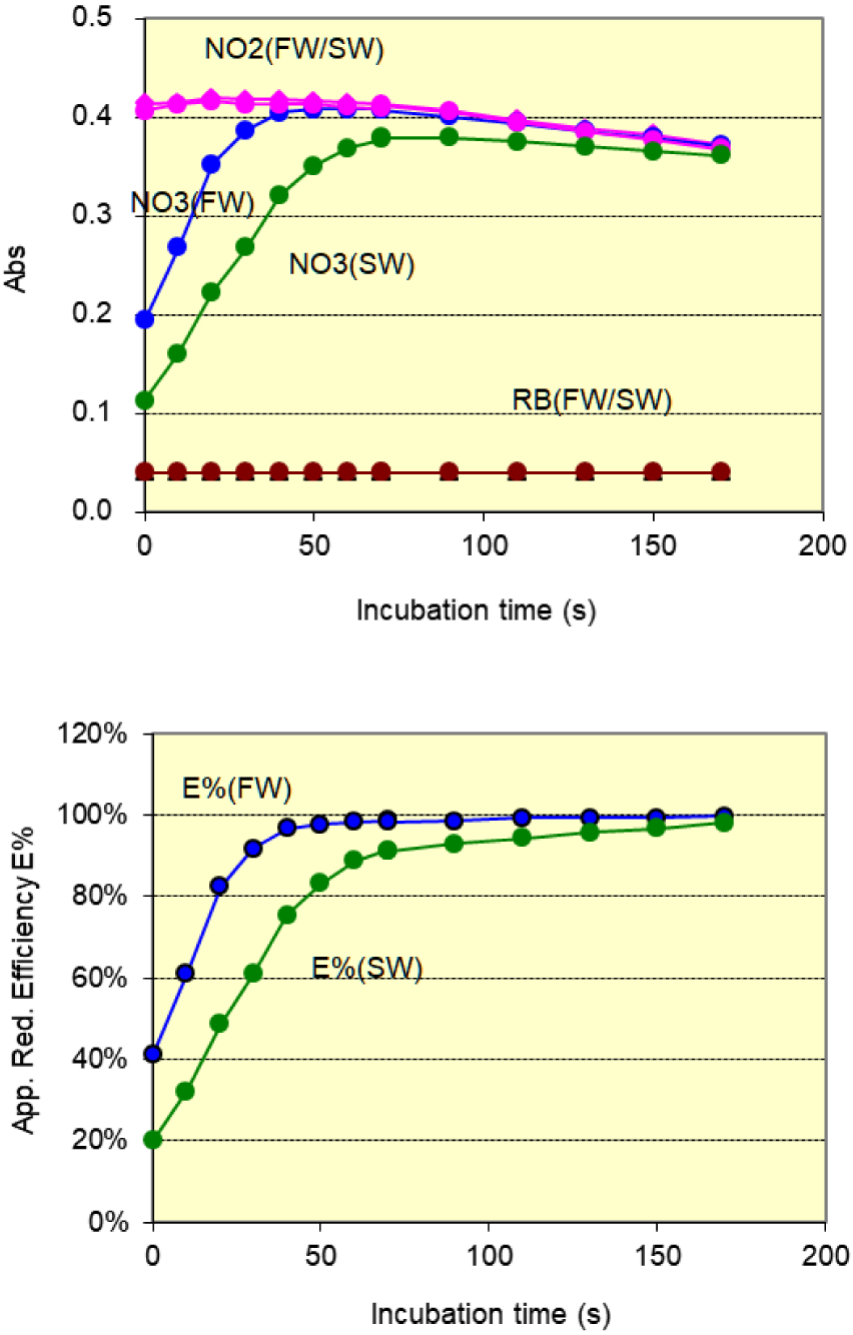
Effect of incubation time on (up) the absorbance of reagent blank, nitrite, and nitrate solutions at an incubation temperature of 90 °C and (down) apparent reduction efficiencies in freshwater and seawater media. Colors represent: pink – 10 μM nitrite in freshwater and seawater; blue – 10 μM nitrate in freshwater; green – 10 μM nitrate in seawater; orange – reagent blank.

The reagent blank remained stable, with absorbance values were in the range of 0.040 ± 0.001 (AU) for both freshwater and seawater. The nitrite signal reached a steady state for incubation time from 20 to 60 s, and was apparently decreased when incubated for more than 70 s.

For nitrate samples in freshwater medium, the reaction was incomplete at shorter incubation times (<40 s), but the apparent reduction efficiency progressively increased, reaching approximately 99% at 70 s and nearly 100% at 90 s. In seawater medium, the apparent reduction efficiency reached 92% at 70 s, increasing slightly to 93% at 90 s, 95% at 130 s, 97% at 150 s, and 98% at 170 s. Although an efficiency greater than or equal to 100% could be achievable with longer incubation times, this was not intended, as the increase was partially due to the fading of the pink azo dye generated from nitrite.

To balance analytical efficiency, color fading, and throughput in automated operation, a 70-s incubation time was selected.

### Recommended Instrumental Settings

Based on the results of the above investigations, the following operational criteria are recommended for the StepFA manifold:

#### Incubation Temperature

The incubation temperature is favorably set at 90 °C. This facilitates the subsequent cooling process to bring down quickly temperature to less than 60 °C when the sample section arrives the flow cuvette. The fading of the pink azo dye will be effectively reduced (judging from the flat-top of a peak). Too high incubation temperatures (95–99 °C) could also result in more tiny bubbles to form in the coil. It should be noted that the initial temperature of sample can be influential. For cold or thawed water samples, they should be left in the laboratory for several hours to equilibrate with room temperature before measurement.

#### Vanadium Chloride Concentration

A freshly prepared, well-shaken, and filtered 3.2% (w/v) VCl_3_ solution is recommended, and it provides a consistent reagent blank of approximately 0.040 AU. Lower concentrations result in incomplete nitrate reduction, while higher concentrations increase the reagent blank undesirably.

#### Incubation Time

The incubation period is fixed at 70 s within a total measurement cycle of 120 s. This cycle also includes 10 s for sample loading, 10 s for delivery, 15 s for detection, and 15 s for flushing.

These setting yield an apparent reduction efficiency of ∼99% in freshwater and ∼92% in seawater—sufficient for most routine applications, provided that precision and calibration curve linearity are maintained. It should be also noted that the temperature along the entire tubular channel is not uniform, as hot and relatively cold liquids alternately pass through the detector in a stepwise manner. Although there was no significant variation on absorbance in the temperature range between 40 and 60 °C, it is still recommended that the system be kept in continuous operation, running in a repeating mode every 130 s (10 s interval between two measuring cycles), with samples manually and sequentially fed into the system.

### Performance and Reproducibility

Based on the above conditions, the performance of the StepFA manifold was demonstrated by repeating measurements on a nitrite/nitrate-depleted seawater spiked with 10 μM of nitrate for 1-h period (Figure 8). The average reagent blank was 0.040. The absorbance readings for 23 measurements ranged from 0.383 to 0.387, demonstrating excellent stability and precision, with relative standard deviations (RSD) well below 0.5%. The standard deviation of the reagent blank was less than 0.001 AU, which is considered the minimum resolution of the method, corresponding to a concentration of approximately 0.03 μM. The detection limit was estimated to be three times that value, or 0.1 μM nitrate.

**Figure 8:**
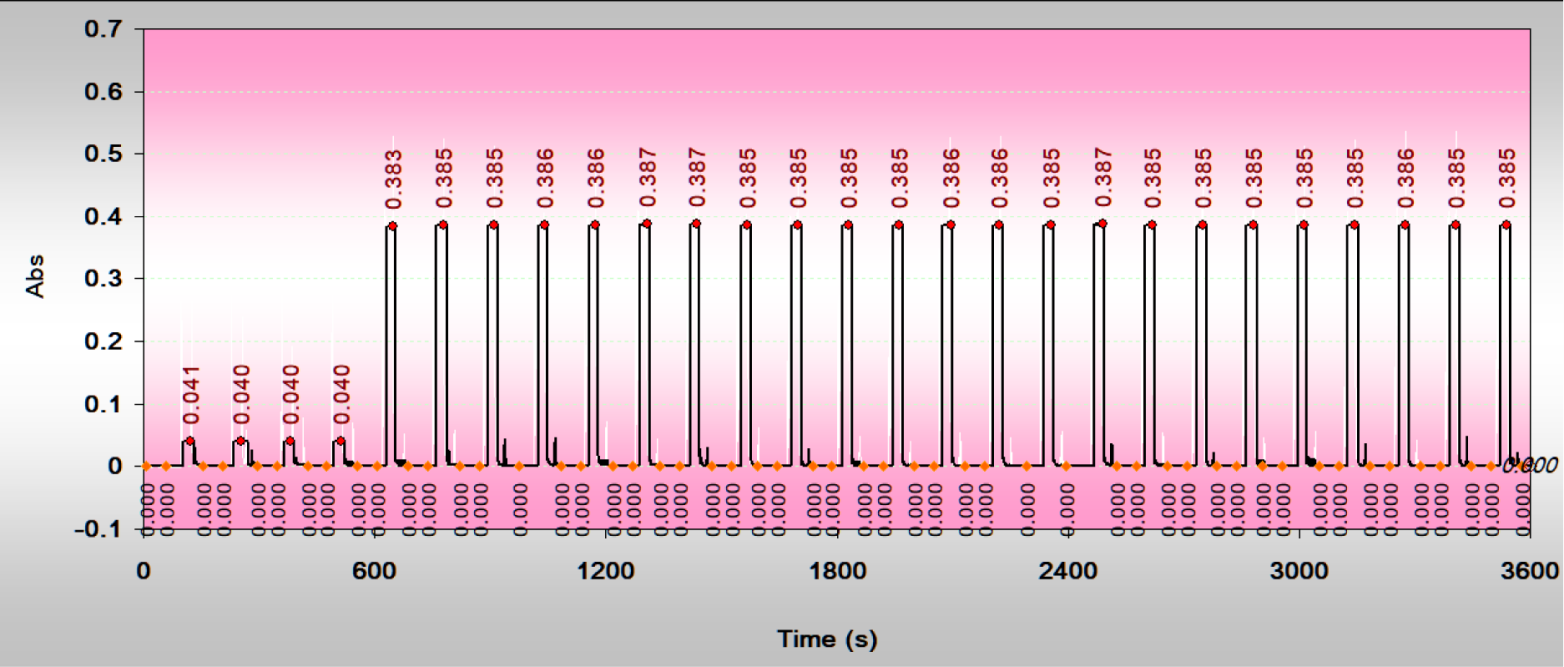
Typical recorder traces for repeated measurements of a seawater sample. The first four peaks represent signals for seawater (reagent blanks), while the subsequent peaks correspond to the same seawater spiked with 10 μM nitrate. Each measurement cycle lasted 120 s, with a 10-s interval between cycles. Large air-section spikes were filtered, but some small random bubble spikes remained. The baseline was checked after each measurement or every 50 s. The absorbance values for the peaks were automatically labeled by the software. All peaks exhibit flat-top shapes resembling histograms, indicating halted reactions and stable signal plateaus.

### Calibration and Quantification

A wide calibration range (0–50 μM) for the pink azo dye was established using nitrite and nitrate standards prepared in distilled water and filtered surface seawater (salinity ∼33) (Figure 9). The incubation time was fixed at 70 s, and the incubation temperature was set at 90 °C. Reagent blank values remained consistent across both media, ranging from 0.039 to 0.041 AU.

**Figure 9:**
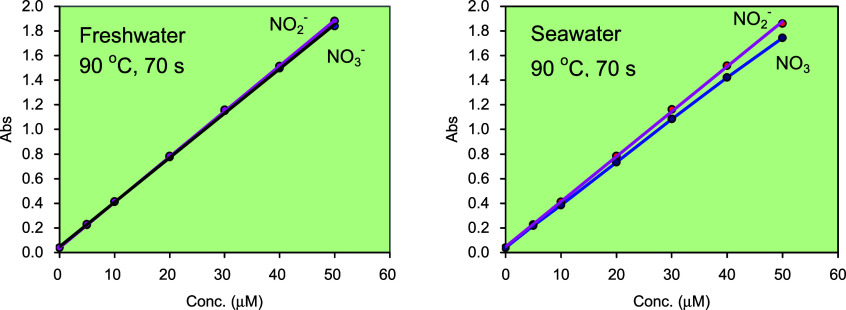
Calibration curves of nitrite and nitrate prepared in distilled water and seawater at an incubation temperature of 90 °C for 70 s, covering the concentration range 0–50 μM. For nitrite measurements, the slopes are 0.0376 μM^– 1^ for both freshwater and seawater media. As for nitrate measurements, the slopes are 0.0373 μM^– 1^ in freshwater and 0.0346 μM^– 1^ in seawater, corresponding to apparent reduction efficiencies of 99% and 92%, respectively.

For nitrite, the calibration slope was 0.0376 μM^–1^ at concentrations below 20 μM, decreasing very slightly by ∼1% at a higher concentration of 50 μM, with no significant difference observed between freshwater and seawater.

For nitrate, the slope was 0.0373 μM^–1^ in freshwater and 0.0346 μM^–1^ in seawater at concentrations below 20 μM, corresponding to apparent reduction efficiencies (*E*%) of 99.2% and 92.0%, respectively. At higher concentrations (30–50 μM), the efficiencies declined slightly by ∼1.5% for both media. Despite these small variations at high concentrations, those calibration curves can be regarded as linear.

In routine analysis, the observed absorbance reflects the combined contribution of pink azo dye produced from both originally existing nitrite and nitrate-derived nitrite. Therefore, the raw result, denoted as [NO_2+3_], is calculated from the raw absorbance as follows:

$$\left[NO_{2+3}\right]=\left(\mathrm{Abs}‐RB\right)/\mathrm{slope}$$

Where RB is the reagent blank, and the slope refers to nitrate standards. The nitrate concentration is then calculated by subtracting the contribution of nitrite and correcting for the reduction efficiency (E%):

$$\left[N{O_{3}}^{‐}\right]=\left[NO_{2+3}\right]‐\left[N{O_{2}}^{‐}\right]/E\%$$

Here, [NO_2_^–^] the nitrite concentration should be measured independently.

For most freshwater measurements (such as river, lake, rainwater, groundwater etc.), since the apparent reduction efficiency *E*% achieved by the StepFA system is sufficiently close to 1, the nitrate concentration may be obtained by directly subtracting the nitrite value. In ocean environment, nitrate concentrations are typically much higher than nitrite concentrations, rendering the correction for nitrite negligible in many cases.

The only potentially problematic scenario arises in the analysis of estuarine waters, where samples may exhibit a wide range of salinities, and both nitrite and nitrate may coexist. In such cases, users may opt to apply an empirical correction. This involves establishing a relationship between salinity and the apparent reduction efficiency using the following equation:

E%=E%(freshwater)×(1‐fS)

Where *f* is an empirical constant, *S* the salinity (0–35). In this work, we have tested diluted seawater at different salinities, giving an empirical *f* value of 0.002 at an incubation temperature of 90 °C.

### Accuracy Check

A certified reference material (Kanso CRM: RMNS for nutrients in seawater, Kanso Technos Co. Ltd., Japan) was tested using the proposed method. The lot number was CS-1790, with certified values of 0.18 μM for nitrite and 16.56 μM for nitrate. Triplicate measurements for [NO_2_^–^ + NO_3_^–^] were 16.88, 16.88, and 16.90 μM, with an average value of 16.89 μM. After correcting for nitrite, the average nitrate concentration found was 16.69 μM, corresponding to a recovery of 100.8%.

### Reagent Consumption and Stability

In the proposed method, each measurement consumes approximately 0.45 mL of each reagent. A 100 mL portion of reagent is sufficient for 180 measurements (6 h). Even though the vanadium reagent remains effective for over a week without special handling, the apparent reduction efficiency may drop significantly. It is explained that the vanadium solution is subject to oxidation if left open to air. For this reason, it is recommended that the vanadium reagent should be prepared daily.

## Conclusion

The proposed system offers a promising approach for nitrate measurement by employing a step-flow autoanalyzer (StepFA)^21^ with an additional stop-over incubation step, which traps the sample in a heating coil, followed by a delivery step that cools the sample before it reaches the detector. These modifications enable the StepFA manifold to effectively perform automated nitrate determination via online vanadium reduction. Heating at 90 °C for 70 s efficiently promotes the vanadium reduction reaction, while the cooling step stabilizes the pink azo dye, prevents color fading. Experimental results show that the apparent reduction efficiency reaches ∼99% in freshwater and ∼92% in seawater. Although the efficiency in seawater is comparatively lower, it does not adversely affect the precision or linearity of the calibration. Calibration curves remain nearly linear up to 50 μM, with a precision better than 0.5% (RSD) and a detection limit of 0.1 μM. A high throughput of 25–30 samples per hour can be readily achieved. The system operates without the need for a carrier flow or injector, and detection occurs under static conditions, thereby eliminating Schlieren effects and carryover issues. Furthermore, inevitable air bubbles no longer pose a problem. Large spikes caused by air sections can be easily filtered out using spreadsheet software, while small, random bubbles do not interfere with detection under nonflowing conditions.

For users without access to a computer for data acquisition or a screen for real-time display, the StepFA manifold can still operate manually in a discrete mode. This involves turning off the autoflush function, following the same procedure, recording the absorbance approximately 100 s after loading, and then pressing the manual flush button to clean the tubular channel.

This study demonstrates a robust, low-cost, versatile, and user-friendly analytical system capable of high-throughput, precise nitrate determination, providing a practical solution for routine nutrient monitoring in both freshwater and marine environments.
